# The Effect of Stacking on the Accuracy of 3D-Printed Full-Arch Dental Models

**DOI:** 10.3390/polym14245465

**Published:** 2022-12-13

**Authors:** Olan Hartley, Tanvi Shanbhag, Derek Smith, Antonio Grimm, Ziad Salameh, Santosh K. Tadakamadla, Frank Alifui-Segbaya, Khaled E. Ahmed

**Affiliations:** 1 School of Medicine and Dentistry, Griffith University, Ian O’Connor Building (G40), Gold Coast, QLD 4215, Australia; 2 Advanced Design and Prototyping Technologies Institute, Griffith University, Gold Coast, QLD 4215, Australia; 3 Faculty of Dental Medicine, Lebanese University, Beirut 1107, Lebanon; 4 Department of Rural Clinical Sciences, La Trobe Rural Health School, La Trobe University, Bendigo, VIC 3550, Australia; 5 Violet Vines Marshman Centre for Rural Health Research, La Trobe Rural Health School, La Trobe University, Bendigo, VIC 3550, Australia

**Keywords:** 3D printing, stacking, trueness, precision, accuracy, dental models

## Abstract

The objective of this study was to assess the effect of stacking on the dimensional and full-arch accuracy of 3D-printed models, utilising a standardised assessment methodology. A previously validated methodology involving a standard tessellation language image (STL) reference model, comprising seven spheres on a horseshoe base resembling a dental arch, was used. Six 3D-designed STL models were prepared, optimised, and stacked horizontally using 3D Sprint software. The stacking file was transferred to the NextDent 5100 printer to build the physical models. To assess accuracy, a coordinate measuring machine (CMM) measured the diameter of the spheres $\left(n=210\right)$, and twenty-one vectors extended between the centres of each of the seven spheres (*n* = 630). When compared to the reference model, significant differences were observed for dimensional (*p* = 0.006) and full-arch accuracy (*p* = 0.006) for all stacked models. Additionally, significant differences were observed between the stacked models for the dimensional accuracy between the posterior (*p* = 0.015), left posterior (*p* = 0.005) and anteroposterior (*p* = 0.002). The maximum contraction was observed in the fourth stacked model, which demonstrated the highest median deviation and least precision within the full-arch (MD = 666 μm, IQR = 55 μm), left posterior (MD = 136 μm, IQR = 12 μm), posterior (MD = 177 μm, IQR = 14 μm) and anteroposterior (MD = 179 μm, IQR = 16 μm) arch segments. In general, the anterior and left posterior arch segments recorded the highest contractions with a median deviation of 34 μm and 29 μm, and precision of 32 μm and 22 μm, respectively. Statistically significant differences were observed between the stacked models in terms of dimensional accuracy that were within clinically acceptable thresholds. The greatest contraction was noted in the fourth model, displaying the least full-arch accuracy compared to the other models. Stacked, additively manufactured, full arch models are a viable alternative for diagnostic, orthodontic, and single-unit prosthodontic applications. In contrast, caution should be exercised when utilising stacked models for full arch high accuracy prosthodontic applications. Further research is needed to assess the impact of additional variables including different printers and resins.

## 1. Introduction

The recent emergence of digital dentistry, including the adoption of hybrid or fully digital workflows in clinical practice, seeks to expedite the delivery of accurate and efficient dental care. Three-dimensional printing (3DP), is a component of the digital workflow commonly used for printing dental models [1], with practical applications across numerous dental disciplines including prosthodontics [2]. Whilst there are various 3DP technologies, the most established ones are photopolymerisation-based stereolithography (SLA) and digital light processing (DLP) [1,3,4,5].

Additive manufacturing may involve the use of different polymeric materials in the form of powder, filaments, or sheet; yet, in dentistry, the most common manufacturing approach employed relies on the polymerisation of photo-sensitive resins with UV or visible-light using DLP or SLA technologies. Both SLA and DLP are versatile technologies and can be used with a variety of photosensitive polymers, either as a closed or an open system. The use of photo-sensitive resins in DLP and SLA does, however, entail the need for additional post-curing processing that may involve washing, drying, and light-curing, with or without nitrogen, of manufactured parts post-printing [6]. The DLP technology works by photopolymerising entirety of each x-y layer at once [6], as the photosensitive liquid resin is exposed to a selectively masked light source [1,2]. SLA is one the earliest and, currently, the most widely utilised printing method in dentistry [7]. In contrast to DLP, in SLA, specific regions per x-y layer of the light-sensitive polymer are successively photocured before dropping the build platform in the *z*-axis [8,9]. Accordingly, the ability of DLP to simultaneously photopolymerise all regions within a printing layer offers a significant advantage regarding the efficiency of the printing cycle through the significant reduction in the manufacturing time.

Whilst the production of 3D-printed models offers a streamlined clinical workflow, the cost [3,10] and printing time may impede the practicality of adopting the digital workflow into clinical practice. Initially, digital impressions are ten times more expensive than the conventional impressions, only equating in cost after 3.6 years of use [11] and the cost of a single 3D-printed model can range from $2 to $9 [12]. According to Li Hsin, Granatelli [6] and Emir and Ayyildiz [12], the printing time for two models varied from 1 h and 48 min to 4 h, depending on the printing system used. To address this, the stacking of dental models has been proposed to increase the 3DP fabrication efficiency, maximise the utilisation of the printer’s build space, and reduce the printing time and material cost [13,14,15,16]. Stacking of objects within a single, continuous, bulk print can potentially reduce the consumption of resources by minimising the support-material waste, reducing labour associated with multiple prints, preparation, and post-processing procedures. This technique may also increase productivity by utilising non-working hours [14].

Full-arch dental models require accuracy in the depiction of the stomatognathic system for effective diagnostic and restorative use [1,17,18]; including the construction of surgical implant guides [2] and multi-unit fixed partial dentures (FPDs) [19], orthodontic treatment planning [20] and the construction of removable prosthodontic prostheses [21]. Despite this, there appears to be limited evidence on the dimensional accuracy of stacking for dental applications. According to Tsolakis, Gizani [22], models with great clinical acceptability for orthodontic purposes can be obtained through various 3DP technology. Currently, the software is designed for the vertical stacking of orthodontic models, which require lower clinical acceptability thresholds (up to 500 μm) in comparison to prosthodontic models (up to 200 μm) [1]. As such, this study aimed to assess the impact of stacking on the dimensional accuracy of 3D-printed full-arch dental models, for prosthodontic application, obtained using DLP technology. To the authors’ knowledge, no studies have investigated the effect of stacking on the dimensional accuracy of full-arch 3D-printed models.

## 2. Materials and Methods

### 2.1. Reference Model

A reference STL file of a model that comprises seven spheres (approximately 10 mm diameter) embedded in a 10 mm thick horseshoe-shaped base was designed using Solidworks (Dassault Systeme, Velizy Villacoublay, France) to mimic the dental arch dimensions, fitting into a medium-sized dental impression stock tray. Surface-matching software (Geomagic Control X, 2014; 3D Systems, Rock Hill, SC, USA) was used to confirm the initial measurements of the STL file including the diameter of each of the seven spheres and the vectors extending between the hypothetical spheres’ centres. The application and full details of this methodology for assessing dimensional and full-arch accuracy of 3DP models has been published elsewhere [6].

### 2.2. Manufacturing 3D-Printed Full-Arch Dental Models

**Figure 1:**
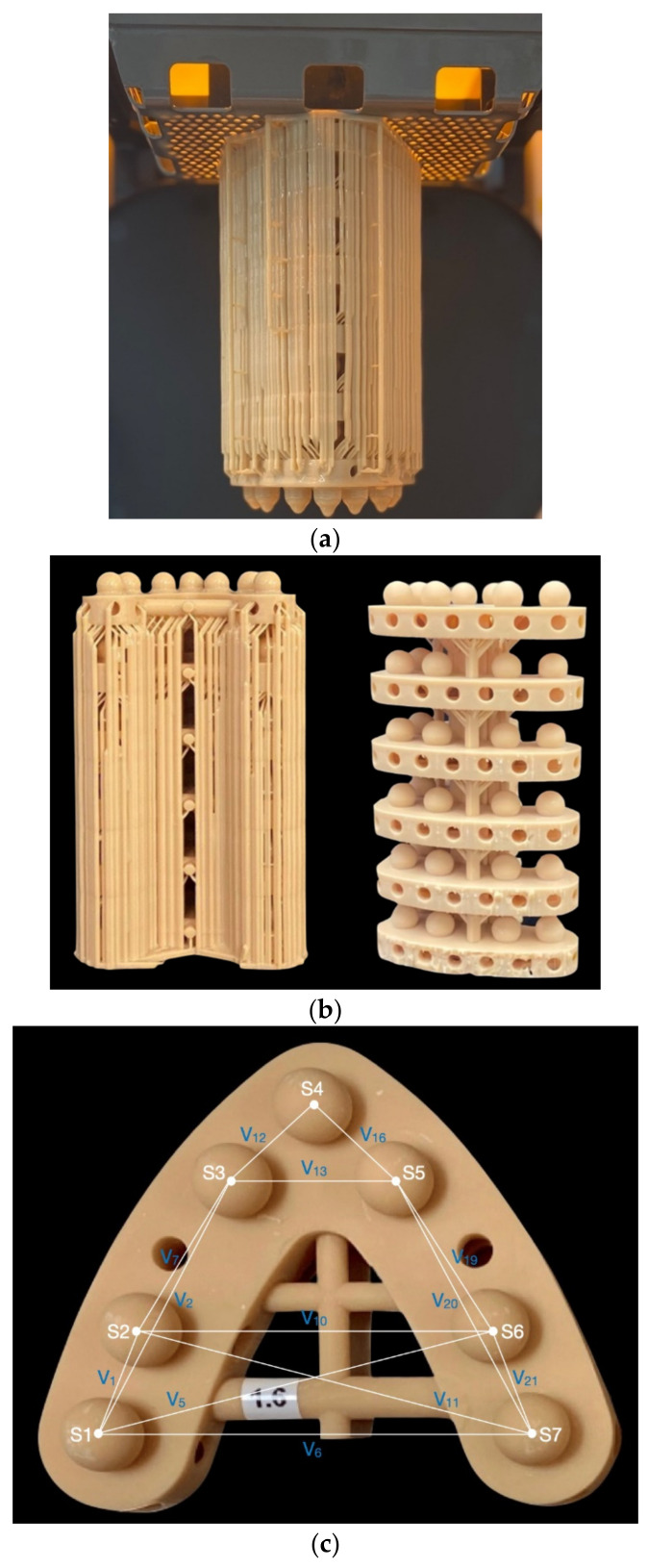
(**a**) Six-tier stacked models with support structures centred on the build plate.(**b**) demonstrates the design of each model with struts and holes for support to minimise warpage. (**c**) shows the anterior and posterior connection bar, with spheres (S), and a representation of the vectors (V) extending between the spheres’ centre to measure full arch and arch-segment accuracy. The accuracy of the anteroposterior arch segment was measured through vector 3 (V_3_), extended between sphere 1 (S1) and sphere 4 (S4), and V_18_, extended between S4 and S7.

In addition to the base diameter of 10 mm, a cross-arch bar was placed to reduce the incidence of warpage, which was observed during the pilot printing (Figure 1a). To optimise the material usage and ensure complete curing, struts and holes were incorporated into the base (Figure 1b). The support structures were generated with the prosthodontic function on the 3D Sprint software and were removed from the ball surfaces at that stage, prior to manufacturing (Figure 1c). The NextDent 5100 (3D Systems, Rock Hill, SC, USA) 3D printer, which utilises DLP 3DP technology was selected. 

To translate the reference model to the NextDent 5100 printer the STL file was imported into the 3D Sprint Basic Software (3D Systems, Rock Hill, SC, USA). The prosthodontic build style with orthodontic supports was utilised to design the six-tier stacked models, with the base oriented at 0 degrees to the build platform. The *z*-axis resolution was standardised and set at 50 μm. All samples were additively manufactured using a manufacturer-validated ethoxylated bisphenol A dimethacrylate photopolymer (NextDent Model 2.0. Vertex-Dental BV, Soesterberg, Netherlands, LOT: WW243N01) with the NextDent 5100 printer.

For the NextDent 5100 printer, the external supports were removed to facilitate postprocessing then the printed models were manually rinsed in >99% ethanol (Thermo Fisher Scientific, Victoria, Australia) for three minutes and then manually agitated in a clean ethanol bath for another two minutes, for no longer than a total of five minutes, as per manufacturer guidelines. The printed parts were air-dried at room temperature for 15 min to ensure they were free of ethanol residue prior to post-curing. Post-curing was performed by placing the models in a pre-heated LC-3DPrint box for 10 min (NextDent, Soesterberg, Netherlands, and thereafter, all remaining internal supports were removed. 

### 2.3. Assessment of Accuracy

The initial measurements, obtained from the reference STL model, comprised seven sphere diameter measurements and 21 vector measurements extending between the spheres’ hypothetical centres. Five batches with six models per build $\left(n=30\right)$ were printed (Figure 1b,c) to assess dimensional $\left(n=210\right)$ and full-arch $\left(n=630\right)$ accuracy. Measurements were obtained within two weeks of printing using a coordinate measurement machine (CMM, Absolute Arm 7-Axis, Hexagon, Cobham, UK) with a 3 mm ruby tip and 50 mm long probe, calibrated in accordance with the International Organization for Standardisation [23] ISO 10360-12: 2016 standard, with an established error of 5 μm. Calibration, as per the standard, involves the probing of a sphere in two locations, 25 points per location, and measuring a bar in seven distributed locations of known length. PolyWorks Inspector (Innovmetric, Québec, QC, Canada) was used to establish an area in space, from six circumferential measurements around the base of the model, obtained using the CMM. The spheres’ dimensions where obtained, through nine-point measurements including one point at the top-centre of the sphere and four circumferential measurements above the equator and four below the equator. The measurements were sequenced from $S_{1}$ to $S_{7}$, with a cartesian axis created in the identified centre of $S_{1},$ after combining the location of $S_{1}$ with the line vector from $S_{1}$ *to* $S_{7}$. The sphere function in Polyworks Inspector was then used to calculate the diameter of the spheres, with the 21 vector measurements, obtained using the hypothetical centre of each sphere. All measurements were obtained by the same operator in a temperature-controlled room (degrees; pressure), with the models stored in the same environment.

The dimensional accuracy of the printed models was assessed through the spheres’ diameter measurements in comparison to reference. The full-arch accuracy of the printed models was assessed through the combination of the 21 vectors calculated between the hypothetical spheres’ centres compared to the reference (Figure 1c). Through the combination of various cross-arch vectors, anterior ($V_{12},\;V_{13},\;V_{16}$), anteroposterior ($V_{3}$, $V_{18}$), posterior ($V_{5},\;V_{6}$, $V_{10}$, $V_{11})$, right posterior ($V_{19}$, $V_{20}$, $V_{21}$), left posterior ($V_{1}$, $V_{2}$, $V_{7}$), the pattern of changes across the respective arch segments were assessed.

### 2.4. Statistical Analysis

This study utilised the ISO standards, which specifies that both trueness and precision measurements are required to quantify the accuracy of 3D models [23]. To determine the dimensional accuracy, the stacked 3D-printed models were assessed for their comparative trueness, the deviation in measurement of the printed model from the reference (quantified by systemic errors), and precision, which refers to the differences between repeated prints (quantified by random errors) [24]. In this study, the median represented trueness, while the interquartile range represented the precision.

Normality of the data was assessed using the Shapiro–Wilk test. Median deviation/error was used to demonstrate the trueness while, interquartile range was considered as a measure of precision while presenting a dimensional and full-arch error. As the data were normally distributed, differences in dimensional error, full-arch efficacy and segment-wise efficacy between the different stacking layers was assessed using one-way ANOVA. Bonferroni post hoc tests were used to determine differences between different stacking layers. Un-paired *t*-tests were used to evaluate the differences between the left and right posterior arch segments and anterior and posterior arch segments. The IBM SPSS statistics software was used to conduct the statistical analysis with a 0.05 significance level. 

## 3. Results

**Figure 2:**
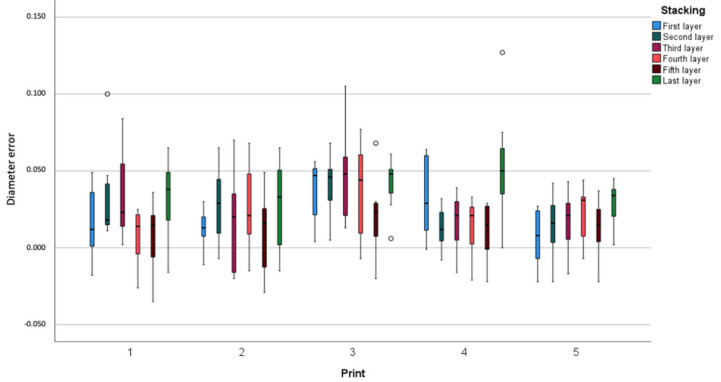
Dimensional error (diameter error of the spheres) in relation to stacking (model) and print. Measurements in millimetres.

A total of five prints of the six-tiered stacked models were obtained from two 1 L bottles of NextDent Model 2.0 photopolymer, and the printing time was 2 h and 57 min. Although one-way ANOVA showed significant differences (*p* = 0.006) between the stacked models for dimensional error, post hoc analysis demonstrated that the only significant difference for dimensional error was between the fifth and sixth model. The fifth layer demonstrated the highest trueness (16 µm) with an increased precision (33 µm), compared to the sixth layer which had the lowest trueness (38 µm) and a decreased precision (36 µm) (Table 1 and Figure 2).

**Figure 3:**
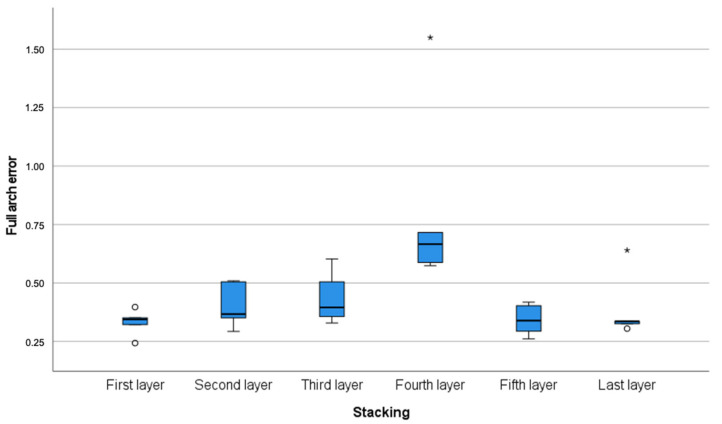
Full-arch error (trueness and precision) calculated through the vectors extended between the centres of the spheres according to stacking layers. Measurements in millimetres. * Denotes outliers.

**Figure 4:**
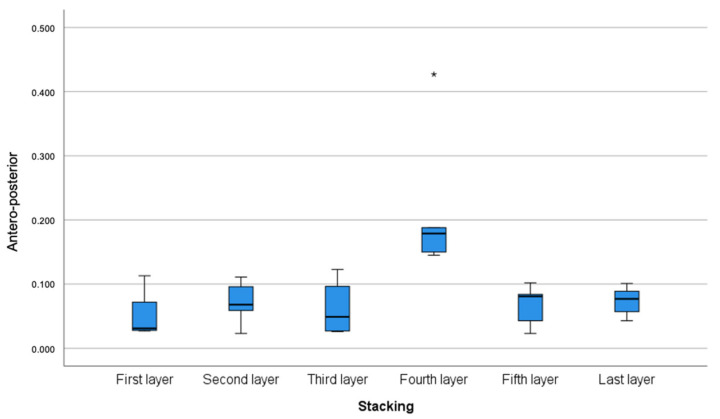
Anteroposterior segment arch error (trueness and precision) calculated through the vectors extended between the centres of the spheres (vectors $V_{3}$, $V_{18}$) according to stacking layers. Measurements in millimetres. * Denotes outliers.

Overall, the fourth model demonstrated the most shrinkage in relation to the reference model and appeared to be the least dimensionally accurate. Significant differences (*p* = 0.006) for the full-arch accuracy were observed between the fourth model and all other models, excluding the third model (Figure 3). Accordingly, the fourth model demonstrated the greatest full-arch contraction with the least trueness and precision of 666 μm and 55 μm, respectively. Similarly, in the anteroposterior (vectors $V_{3}$, $V_{18}$) arch segment, statistically significant differences (*p* = 0.002) were observed between the fourth model and all other models. A substantial amount of contraction was noted on the fourth model, with a trueness of 179 μm and precision of 16 μm (Table 2 and Figure 4).

Consistently, significant differences (*p* = 0.005) were observed for the left posterior (vectors $V_{1}$, $V_{2}$, $V_{7}$) arch segment between the fourth model and most of the models; with the fourth model displaying the most shrinkage with a higher median deviation error (136 μm) and interquartile range of 12 μm. No differences were observed for the right posterior (vectors $V_{19}$, $V_{20}$, $V_{21}$) arch segment between the stacked models (Table 2). However, significant differences (*p* < 0.001) were seen between the left and right posterior arch segments in relation to the dimensional accuracy of all models, regardless of the batch. The left posterior arch segments were less dimensionally accurate compared to the right posterior arch segments. More shrinkage was detected in the left posterior arch segment compared to the right side with a trueness of −6 μm and 29 μm and precision of 21 μm and 22 μm, respectively (Table 3).

Statistically significant differences (*p* = 0.015) were observed between the fourth model and the first and fifth models in the posterior ($\mathrm{vectors}\;V_{5},\;V_{6}$, $V_{10}$, $V_{11})$ arch segments. Additionally, the fourth model demonstrated the greatest posterior cross-arch shrinkage with a trueness of 177 μm and precision of 14 μm. No statistically significant differences were observed for the anterior (vectors $V_{12},\;V_{13},\;V_{16}$) segment between the stacked models (Table 2). Contrastingly, there were statistically significant differences (*p* < 0.001) between the anterior and posterior arch segments in relation to the dimensional accuracy of all the models, regardless of the batch. A greater magnitude of contraction was noted in the anterior segment compared to the posterior segment. The anterior arch segment demonstrated a lower trueness (34 μm) but higher precision (32 μm) than the posterior arch segment, which had a trueness of 13 μm and precision of 60 μm (Table 4).

## 4. Discussion

There is limited literature available investigating the accuracy of stacking in additive manufacturing and, to the authors knowledge, this is the first study investigating the impact of stacking on the accuracy of dental models.

The validated assessment protocol employed in this study obtained reference measurements using a CMM and inspection software as a gold-standard, utilising both metrology and 3D linear measurements. The methodology was previously utilised by Li Hsin, Granatelli [6] for evaluating the impact of storage on the dimensional accuracy and stability of full-arch 3D-printed models in SLA and DLP printers. The main advantage of the methodology is its ability to avoid measurement errors associated with manual calliper measurements as well as iterative closest point registration [6]. Moreover, all measurements were obtained by a single operator within two weeks post-printing of samples to ensure standardisation, minimise interoperator errors, and dimensional changes exhibited by the 3D-printed models due to storage [6]. 

To reduce material use and optimise the printing time, a hollow model was chosen which has been demonstrated to have no impact on the accuracy of full-arch models [1,22]. The reference model was designed with the optimal geometric configuration, comprising perfect spheres, to effectively avoid the superimposition of certain anatomical features, reducing errors and enabling the reproducibility of measurements. Bilaterally, the posterior spheres $\left(S_{1},\;S_{2},\;S_{6},S_{7}\right)$ represent the premolars and molars and the canine-to-canine segment is represented anteriorly $\left(S_{3},\;S_{4},\;S_{5}\right)$. This is designed to account for the fact that creating reproducible reference points on dentate models for measurement is deemed challenging [17], as teeth are not geometrically equal.

There are several factors that may introduce errors in the accuracy of the 3D-printed full-arch dental models, including the slicing method [25,26], material composition and printer parameters [10], polymerisation shrinkage [27,28], post-processing procedures [26,28,29,30] and storage [6,19,31]. Moreover, the accuracy of the printed model can be affected by the manufacturing parameters such as the layer thickness, build orientation, support structure, thermal errors, slice thickness and post-curing [2,10,32]. To minimise the potential of errors in this study, only manufacturer recommended materials—including a validated monomer based on methacrylic ester for sample-printing, pre-set manufacturing parameters, and recommended post-processing techniques were used.

The sample models were centred on the build platform and built at 0 degrees to the x-axis, to maximise the quantity of models that could be stacked in the build volume while maintaining accuracy. In stacking, the orientation and positioning of the models is imperative to optimise the space used in the build vat [15], reducing the build-time and print-height, whilst avoiding collisions [16]. The build-height directly impacts the efficiency and printing cost and as such, 3D models should be packed as closely as possible [13,16]. Stacking multiple models in each print load eliminates multiple start-over printing procedures, incurring fewer material costs and time and has the potential to utilise non-working hours [14], addressing the high-cost and time-consuming printing and post-processing associated with 3DP [10]. Through stacking, a total of six models were printed within 2 h and 57 min. In contrast, previous studies reported a timeframe between 1 h and 48 min to 4 h for the printing of just two models, depending on the printer used [6,12].

Previous studies have concluded that the horseshoe base produces clinically acceptable results, with reduced material use; however, Camardella, Vilella [33] postulated that contraction may occur in the transversal dimension during the post-curing protocol. To address this, a posterior connection bar was utilised to prevent the post-curing contraction of the posterior arch segment, as suggested in previous in vitro studies [29,33]. This is in-line with a recent systematic review by Tsolakis, Gizani [22], which highlighted the accuracy of DLP printed models with a horseshoe base supported with a posterior connection bar to mitigate distortion. The reduced posterior segment contraction observed can be attributed to the addition of this posterior connection bar and struts during the design of the STL file. Nonetheless, significant dimensional shrinkage still occurred in the posterior segment of the samples. This concentration of posterior deviation was identified in a recent systematic review investigating the accuracy of digital versus conventional workflow in prosthodontics with the reported posterior contraction attributed to photopolymer warpage or shrinkage related to the post-polymerisation required for SLA and DLP printing [9].

Recent reviews have identified that the clinically acceptable range of error for prosthodontic applications ranged between 200 μm [1] to <120 μm [9]. Additionally, DLP printed models displayed accuracy within <100 μm [1] with another study finding DLP printed models to have a clinically acceptable 59–150 μm margin of error, rendering the DLP printed models suitable for prosthodontic application, ideally within 2–3 weeks post-printing [6,19]. In this study, whilst the first and sixth models demonstrated the highest full arch accuracy, the full arch accuracy for all models exceeded the clinically acceptable thresholds, with the fourth model displaying the least accuracy possessing the greatest deviations. The full arch accuracy discrepancies noted between the stacked models may be attributed to manufacturing warpage of the internal layers of the build, resulting from distortions during the post-curing process, which is a limitation identified with DLP printers [9]. Accordingly, this limited full arch accuracy presents serious challenges to the reliable use of stacked models for full arch prosthetic rehabilitation. In contrast, the dimensional accuracy, represented by the individual spheres, for all the stacked models was within the clinically acceptable range, with the fifth model displaying the highest dimensional accuracy. This high dimensional accuracy noted indicates that stacked models are a viable alternative for single-tooth restorations. It is worth noting that whilst the sixth printed model within the stack did exhibit the least dimensional accuracy, this was still within the clinically acceptable range. A possible explanation for the observed error could be the minimal layer thickness used during the build construction which may have led to sequential contraction during the layering process. This may have added to the errors observed in the *z*-axis [34], corroborating the study findings of the sixth model in the stacked print demonstrating the least dimensional accuracy. Additionally, the accumulation of residual stress and contraction during polymerisation shrinkage may have contributed to variable dimensional distortion across the models [34].

Numerous constraints exist around the model orientation due to restricted regions, where support structures cannot be placed [14]. Current research on stacking has focussed on optimising the support structures to reduce material usage, cost, post-processing, and print time [14]. Multiple studies have proposed altering the orientation of the support structures to effectively minimise the volume of the support structures [32,35,36,37,38]. The horizontal stacking arrangement facilitated the placement of support structures distant to critical areas, ensuring greater accuracy and reducing post-processing [32]. Additionally, in the case where the support structure is not adequately removed during post-processing or the support structure has resulted in damage to the printed parts, scarring may occur which can affect the quality of the product [39]. To ensure greater accuracy of the CMM measurements, the support structures were removed from the surface of the spheres to prevent surface scarring to ensure measurement validity of critical areas.

Dimensional accuracy for diagnostic use and treatment planning [40], and procedures such as the fitting of indirect restorations to prepared tooth structures [21] is of paramount clinical importance. Accordingly, the stacked models are deemed unsuitable for high precision, full arch applications, irrespective of the model’s sequence within the stack. As such, caution should be exercised when relying on stacked 3D-printed models for prosthodontic application, as more research is necessary to validate the routine use of stacked models for indirect restorative purposes.

Whilst the standardised reference model has facilitated reproducibility in testing, the in vitro nature of this study is a limitation that is shared with similar studies, as the reference model comprises a simple geometrical design. A drawback to this design is the inability to represent the anatomical variations present within the stomatognathic system. Moreover, the slicing software that was used to generate the six-tier stacked design including the support structures has previously only been used for the vertical stacking of orthodontic models, which have more relaxed clinically acceptable thresholds in comparison to prosthodontic models. To address these limitations, future studies are needed to investigate varying support structure configurations and post-processing procedures and their impact of accuracy, print-time, and material-use. Furthermore, a minor procedural error encountered in this study was the incomplete formation of the sixth and seventh spheres observed on the third model of the second print, which may be attributed to a *z*-axis error. Additionally, the use of the DLP 3DP technology and manufacturer recommended photopolymer may not be representative of the results that may be achieved with other widely used printing technologies such as SLA. Further research is needed to determine the accuracy of the 3D-printed models using different printing technologies.

## 5. Conclusions

The findings of the study identified that stacking had a significant impact on the model’s dimensional and full-arch accuracy. The dimensional accuracy of all the models appeared to fall within the clinically acceptable range of error for both diagnostic, orthodontic and single-unit prosthodontic applications. In contrast, the full-arch accuracy of stacked models exceeded the clinically acceptable range for high-accuracy full arch rehabilitation prosthodontic applications, irrespective of their sequence within the printed stack. Further research needs to be conducted to assess the suitability of stacking for prosthodontic models with varying manufacturing parameters to improve its accuracy.

## Figures and Tables

**Table 1:** Dimensional error (median, trueness) of the spheres’ diameter of the stacked models. Precision expressed as interquartile error (IQR). Measurements in millimetres.

	First Layer (A) Median (IQR) (mm)	Second Layer (B) Median (IQR) (mm)	Third Layer (C) Median (IQR) (mm)	Fourth Layer (D) Median (IQR) (mm)	Fifth Layer (E) Median (IQR) (mm)	Sixth Layer (F) Median (IQR) (mm)	***F(p)***	**Post Hoc Using Bonferroni Test**
Dimensional Error	0.019 (0.036)	0.026 (0.03)	0.023 (0.036)	0.021 (0.039)	0.016 (0.033)	0.038 (0.036)	3.403 (0.006)	F > E

**Table 2:** Median error (trueness) of the stacked models for full-arch, left posterior, right posterior, posterior, anterior and antero-posterior arch segments. Precision expressed as interquartile error (IQR). Measurements in millimetres.

	First Layer (A) Median (IQR) (mm)	Second Layer (B) Median (IQR) (mm)	Third Layer (C) Median (IQR) (mm)	Fourth Layer (D) Median (IQR) (mm)	Fifth Layer (E) Median (IQR) (mm)	Sixth Layer (F) Median (IQR) (mm)	*F(p)*	Post Hoc Using Bonferroni Test
Full-Arch	0.344 (0.09)	0.436 (0.20)	0.396 (0.21)	0.666 (0.55)	0.339 (0.13)	0.336 (0.17)	4.414 (0.006)	D > A D > B D > E D > F
Left Posterior	0.076 (0.04)	0.067 (0.03)	0.069 (0.06)	0.136 (0.12)	0.047 (0.04)	0.054 (0.02)	4.613 (0.005)	D > B D > E D > F
Right Posterior	0.087 (0.05)	0.092 (0.04)	0.084 (0.13)	0.114 (0.10)	0.069 (0.03)	0.065 (0.08)	1.109 (0.384)	
Posterior	0.085 (0.06)	0.126 (0.06)	0.093 (0.05)	0.177 (0.14)	0.82 (0.11)	0.103 (0.11)	3.603 (0.015)	D > A D > E
Anterior	0.038 (0.05)	0.069 (0.05)	0.086 (0.07)	0.077 (0.12)	0.062 (0.03)	0.07 (0.04)	1.623 (0.194)	
Antero-posterior	0.031 (0.07)	0.082 (0.05)	0.049 (0.08)	0.179 (0.16)	0.081 (0.06)	0.077 (0.05)	5.638 (0.002)	D > A D > B D > C D > E D > F

**Table 3:** Median error (trueness) of left and right posterior arch segments and precision, expressed as interquartile error (IQR). Measurements in millimeters.

	Right Median (IQR) (mm)	Left Median (IQR) (mm)	*t*	*p* Value
Posterior Arch Segments	−0.006 (0.021)	0.029 (0.022)	11.905	<0.001

**Table 4:** Median error (trueness) of antero-posterior arch segments and precision, expressed as interquartile error (IQR). Measurements in millimeters.

	Anterior Median (IQR) (mm)	Posterior Median (IQR) (mm)	*t*	*p* Value
Antero-posterior Arch Segments	0.034 (0.032)	0.013 (0.06)	7.969	<0.001

## Data Availability

Not applicable.
